# *Liopropomaincandescens* sp. nov. (﻿Epinephelidae, Liopropominae), a new species of basslet from mesophotic coral ecosystems of Pohnpei, Micronesia

**DOI:** 10.3897/zookeys.863.33778

**Published:** 2019-07-11

**Authors:** Hudson T. Pinheiro, Bart Shepherd, Brian D. Greene, Luiz A. Rocha

**Affiliations:** 1 California Academy of Sciences, San Francisco, CA 94118, USA California Academy of Sciences San Francisco United States of America; 2 Association for Marine Exploration, Kaneohe, HI 96744, USA Association for Marine Exploration Kaneohe United States of America

**Keywords:** closed-circuit rebreather, coral-reef twilight zone, reef fish, SubCAS, taxonomy

## Abstract

A new species of the genus *Liopropoma* Gill found on the lower mesophotic coral ecosystem of Pohnpei, Micronesia, is herein described. *Liopropomaincandescens***sp. nov.** differs from its congeners in coloration, number of lateral-line scales, number of pectoral fin rays, body depth, and snout length. *Liopropomaincandescens***sp. nov.** is the 31^st^ species in the genus. It was collected from a small rocky crevice in a steep slope at 130 m depth. Water temperature was 20 °C and benthic habitat was dominated by gorgonians, sponges and tunicates.

## Introduction

Despite significant recent growth on research in mesophotic coral ecosystems (MCEs, 30–150 m depth), exploration at these depths is still yielding high rates of new species discovery worldwide (Rocha et al. 2017, Pinheiro et al. 2018, Pyle et al. 2018, Shepherd et al. 2018a, Arango et al. 2019). Through rebreather technical diving to depths up to 150 m, the Hope for Reefs Initiative of the California Academy of Sciences is advancing the knowledge ca. the biodiversity and ecology of MCEs conducting on average four expeditions per year. MCEs shelter unique communities, and much like their shallow coral ecosystem counterparts, are being affected by overfishing, pollution and climate change (Rocha et al. 2018).

Coral reefs of the Central Pacific are home to a high fish diversity, with over 2,300 species (Kulbicki et al. 2013). Pohnpei, one of the four states in the Federated States of Micronesia, has well-developed shallow coral reefs and MCEs, which also harbor diverse mesophotic fish communities (Coleman et al. 2018, Rocha et al. 2018). Technical diving exploration in Pohnpei MCEs has yielded the recent descriptions of species in the genera *Luzonichthys* Herre (e.g., Copus et al. 2015) and *Tosanoides* Kamohara (e.g., Pyle et al. 2018). Although overall richness decreases significantly along the depth gradient, high betadiversity, driven mainly by species turnover, differentiates shallow from deep reefs in the region (Coleman et al. 2018, Rocha et al. 2018).

The serranid genus *Liopropoma* Gill is a typical inhabitant of MCEs, with the most recent species being described from the Caribbean (Baldwin and Robertson 2014; Baldwin and Johnson 2014). It is characterized by VIII, 11–14 dorsal fin rays, III, 8–11 anal rays, weak ctenoid scales on the body, a complete lateral line (highly arched over pectoral fin) with 44–66 pored scales, and a band of villiform teeth in both jaws lacking canines (Kon et al. 1999). According to Eschmeyer and Fong (2018), *Liopropoma* currently contains 30 recognized species, 22 of which are distributed in the Indo-Pacific region, with a further eight species in the Western and Eastern Atlantic. These authors consider *Liopropomadanae* (Kotthaus) as a doubtful species, almost certainly a synonym of a valid species, described based on juvenile specimens. During our latest expedition to Pohnpei in August of 2017, we discovered a new species of *Liopropoma* at 130 m depth at Ahnd (Ant) Atoll. Here we describe *Liopropomaincandescens* sp. nov. as the 31^st^ species of the genus.

## Materials and methods

The specimen was collected with hand nets during a deep dive using mixed-gas, closed-circuit rebreathers, and brought to the surface alive in the SubCAS submersible fish decompression chamber (Shepherd et al. 2018b). It was placed in a 1 L plastic bag filled with seawater and pure oxygen in equal ratios, packed inside a Styrofoam box with a cardboard outer liner, and transported alive via air cargo to San Francisco, where it was photographed and euthanized following California Academy of Sciences institutional animal care and use committee (IACUC) guidelines. ﻿Counts were performed with a stereo microscope, and morphological characters were measured to the nearest 0.01 mm with digital calipers following the conventions described in Baldwin and Robertson (2014). Body proportions are expressed as percentage of standard length (SL). ﻿Comparative material included *Liopropomaafricanum* (Smith) (CAS 32371), one specimen 37.67 mm SL, from Comoros Islands, collected 25–30 m depth; *Liopropomacollettei* Randall & Taylor (CAS 228952), one specimen 54.55 mm SL, from Honolulu, Hawaii, collected ca. 15 m depth; *Liopropomafasciatum* Bussing (CAS 46579), one specimen 117.30 mm SL, from Galapagos, Ecuador, collected ca. 20 m depth; *Liopropomalatifasciatum* (Tanaka) (CAS 243779), one specimen 54.06 mm SL, from Okinawa, Japan; *Liopropomalongilepis* Garman (CAS 86404), one specimen 145.52 mm SL, from Galapagos, Ecuador, collected from a submersible down to 200 m; *Liopropomamitratum* Lubbock & Randall (CAS 27698), one specimen 41.06 mm SL, from Raroia Atoll, French Polynesia, collected ca. 10 m depth; *Liopropomamowbrayi* Woods & Kanazawa (CAS 56894), one specimen 43.40 mm, from U.S. Virgin Islands; and *Liopropomasusumi* (Jordan & Seale) (CAS 214155), one specimen 52.01 mm SL, from the American Samoa, collected ca. 15 m depth; along with a ﻿revision of the genus by Randall and Taylor (1988). The holotype was deposited at the California Academy of Sciences (CAS) ichthyological collection.

## Results

### Liopropoma incandescens sp. nov.

Taxon classificationAnimaliaPerciformesSerranidae

http://zoobank.org/ADC07533-17B1-4FAC-9C56-67C47D8AB8AD

**Figure 1.**
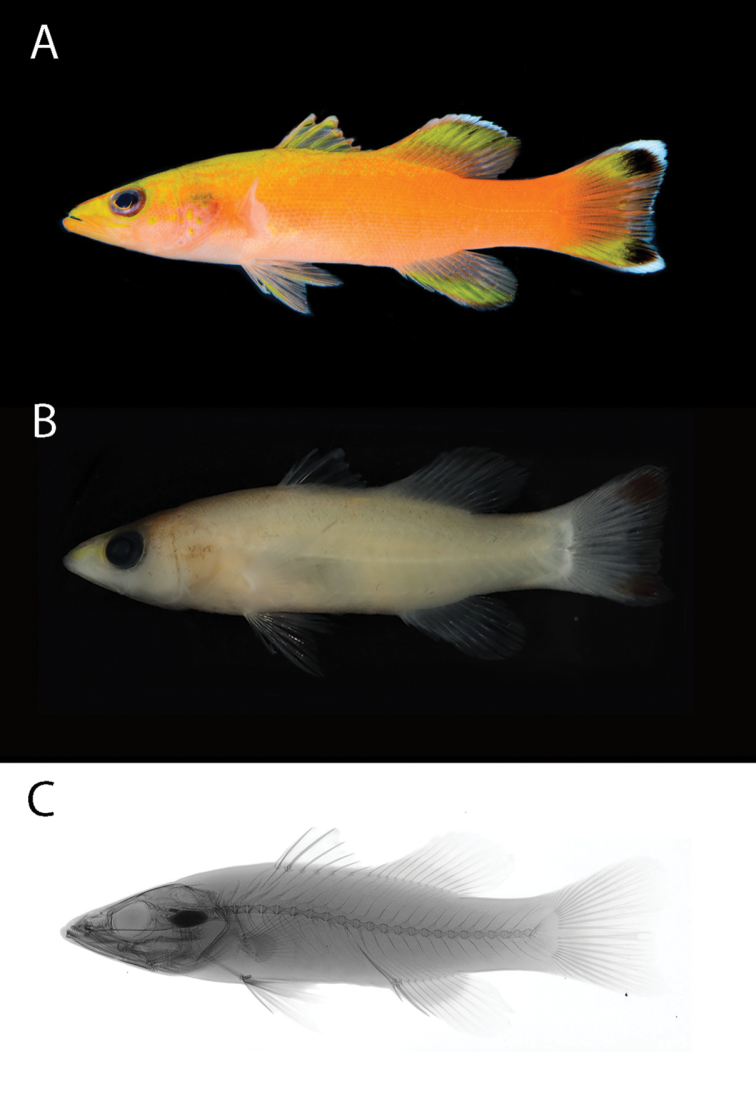
Holotype of *Liopropomaincandescens* (CAS 246199), 54.15 mm SL, collected at a depth of 130 m off Ahnd (Ant) Atoll, Pohnpei, Federated States of Micronesia. Photographs by L. A. Rocha and J. Fong.

**Figure 2.**
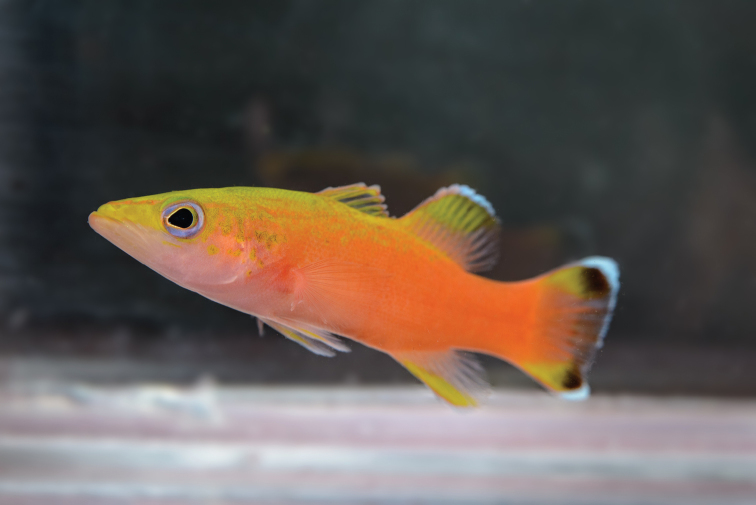
Holotype of *Liopropomaincandescens* (CAS 246199), 54.15 mm SL, shown alive in an aquarium. Photograph by L. A. Rocha.

Figures 1 , 2 Incandescent Basslet

#### Type locality.

Ant Atoll, Pohnpei, Micronesia.

#### Holotype.

CAS 246199, 54.15 mm SL, ﻿Federated States of Micronesia, Pohnpei, Ahnd (Ant) Atoll, west side, 6.75589N, 157.91933E, 29 August 2017, B. D. Greene, hand nets, 130 m depth.

#### Diagnosis.

*Liopropomaincandescens* sp. nov. can be distinguished from all of its congeners by the yellow to orange body color (Figure 1A), with two distinctive black blotches on the upper and lower caudal fin lobes (Figure 1A, B), and by the following combination of characters: dorsal fin VIII,13; anal fin III, 9; pectoral fin 14; total gill rakers on first arch 15; lateral-line scales 62; length of first dorsal-fin spine 5% SL; depth at origin of dorsal fin 22% SL; least depth of caudal peduncle 15% SL; orbit diameter 9% SL.

#### Description.

Dorsal-fin rays VIII, 13 (spines not embedded into the skin, and the last two soft rays are associated with a single complex pterygiophore; Figure 1C); anal-fin rays III, 9 (last two soft rays associated with a single complex pterygiophore); pectoral-fin rays 14, dorsal-fin-most ray unsegmented; pelvic-fin rays I, 5; principal caudal-fin rays 8+7=15; rudimentary and procurrent caudal-fin rays 10+9=19; pored lateral-line scales 62; scales from lateral line to dorsal fin origin 6; scales from lateral line to anal fin origin 22; gill rakers on first arch, including rudiments, 6+9; vertebrae 8+15 (Figure 1C).

Measurements presented as percentage of standard length (SL): body depth at origin of dorsal fin 21.8; body width just behind gill opening 14.9; head length 38; snout length 9.5; orbit diameter 8.6; bony interorbital width 6.4; upper-jaw length 14.7; maxillary length 12.7; least caudal-peduncle depth 14.6; caudal-peduncle length 13.5. Fin lengths: dorsal-fin spines: (I) 4.6; (II) 9.9; (III) 10.7; longest dorsal-fin soft ray the 10^th^, length 17.4; lengths of anal-fin spines: (I) 3.9; (II) 10.1; (III) 11.3; longest anal soft ray the 5^th^, length 18.1; caudal-fin length 23.2; pectoral-fin length 26, fin short, not reaching vertical between anus and dorsal fin; pelvic-fin length 19, fin reaching vertical slightly posterior to base of 5^th^ dorsal-fin spine.

Interorbital region flat; mouth oblique, maxilla almost reaching vertical crossing posterior border of pupil; prominent bony projection on posteroventral corner of maxilla; lower jaw projected. Anterior nostril in thin, membranous tube, situated close to tip of snout; posterior nostril a simple opening, situated close to orbit. Lateral line strongly arched above pectoral fin, highest point below fourth dorsal-fin spine. Trunk covered with ctenoid scales, scales becoming weakly ctenoid anteriorly and cycloid on head. Head fully scaled except over branchiostegal area. Short membrane covered by scales anteriorly to first dorsal-fin spine, six rows of scales covering basal anterior portion of soft dorsal fin, decreasing uniformly to two scales at posterior basal portion of soft dorsal fin. Anal fin with two to five rows of scales basally (more rows between second and fourth spine. Caudal fin almost completely scaled, except for ﻿distal tips of rays. Scales present on pectoral-fin base, pelvic-fin base, and on proximal portion of pelvic fin. Jaw teeth small; upper and lower jaws with bands of villiform teeth, bands slightly wider anteriorly. Vomer oval patch of small teeth. Palatines with several rows of small teeth in long and narrow bands at each side of mouth. Opercle with one conspicuous middle spine. Margin of upper and lower limb of preopercle smooth.

#### Color in life.

Alive and freshly euthanized holotype (Figures 1A, 2) with coloration as follows: snout, top of head and trunk yellow, grading to vivid orange on a diagonal around upper two-thirds of body to caudal fin. Pale pink checks with yellow blotches behind eye, on operculum, and on dorsal-most part of body from head to base of soft dorsal fin. Indistinct orange line from tip of snout, across top of eye, continuing to above preopercle edge. Pupil black with yellow outer margin; eye pale purple and orbit with orange ring along margin. Pale pink-orange to peach-colored throat, continuing below pectoral fins, and across belly. Dorsal fin with yellow-orange tipped spines and mostly translucent inter-spinous membranes; base of soft dorsal-fin rays (ventral third, scaled region) orange; soft dorsal fin central upper region yellow from first to eighth ray; margin of the first soft dorsal-fin ray orange, transitioning to white from second to eighth; dorsal portion between eighth and twelfth soft ray light orange, with no white margin and no yellow. Pectoral fin light orange, yellow anteriorly (first ray). Upper two-thirds of anal fin orange, distal region yellow from second to ninth ray; anal-fin margin white along second to ninth ray with a pale orange sub-border, translucent along tenth to twelfth ray. Central portion of caudal fin with orange rays and membranes, with white pigments in the distal posterior third; upper and lower portions of caudal fin yellow with orange base; two pronounced oval-shaped black spots with posterior white margins, approximately the same size as orbit, on outer upper and lower ﻿caudal lobes.

#### Color in alcohol.

In alcohol (Figure 1B), body light beige, pigment only present on a small patch of yellow on the snout, and the two pronounced dark spots on distal upper and lower caudal fin lobes.

#### Distribution.

*Liopropomaincandescens* sp. nov. is known based on one specimen collected at a depth of 130 m in Ahnd (Ant) Atoll, Pohnpei, Federated States of Micronesia. The lack of records for the species in other MCEs of the Pacific Ocean is probably due to its cryptic habits combined with the lack of sampling at those depths across the wider region.

#### Habitat and behavior.

**Figure 3.**
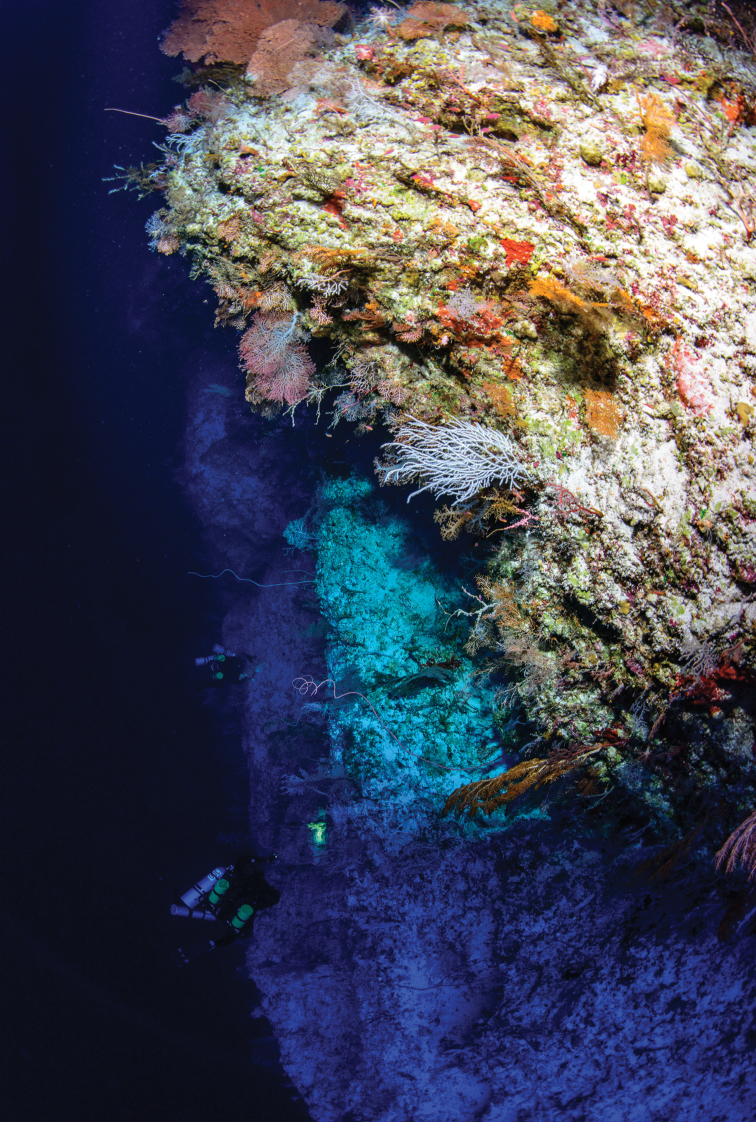
Coral reef wall at a depth of 130 m, the habitat where *Liopropomaincandescens* was discovered in Ahnd (Ant) Atoll, Pohnpei. Photograph by L. A. Rocha.

*Liopropomaincandescens* sp. nov. has a cryptic habit and was discovered and ﻿collected in a small rocky crevice along a steep limestone coral reef drop-off at a depth of 130 m (Figure 3). A second individual (~10 cm length) was sighted in the same area, together with species such as *Tosanoidesannepatrice* Pyle, Greene, Copus & Randall, *Centropygeabei* Allen, Young & Colin, *Odontanthias* sp. and *Roa* sp., but was not collected.

#### Etymology.

The specific name is a noun in apposition from the Latin, *incandescens*, to glow. The vivid yellow to orange incandescent coloration of the species prompted us to select this name.

#### Comparisons.

The color of *Liopropomaincandescens* sp. nov. sets it apart from all other species in the genus: the gradient from yellow to orange and the two black spots on the upper and lower caudal fin lobes are unique. The only other species with black spots on upper and lower caudal fin lobes is *Liopropomacarmabi* (Randall), from the Western Atlantic, which has alternating orange and pink horizontal lines running from the snout to the caudal fin. Moreover, most species of *Liopropoma* have between 44 and 54 lateral-line scales, whereas *L.incandescens* sp. nov. shares a high number of lateral-line scales (62) exclusively with *Liopropomamaculatum* (Döderlein) (Randall and Taylor 1988, Kon et al. 1999, Wirtz and Schliewen 2012, Baldwin and Johnson 2014, Baldwin and Robertson 2014). *Liopropomaincandescens* sp. nov. differs from *L.maculatum* by the lower number of pectoral fin-ray counts (14 vs 15–16), the presence of two black spots on outer upper and lower ﻿lobes of the caudal fin, a more slender body (body depth 4.6 vs 3.2–4 in SL), and shorter snout (4 vs 3.3–3.7 in HL).

## Discussion

The shallow coral reefs of Micronesia are known to shelter a high diversity of reef fishes (Myers 1999, Kulbicki et al. 2013), and the clear waters of central Pacific seem also to favor biodiverse communities at mesophotic depths. However, communities change considerably along the shallow to mesophotic gradient. When studying shallow and deep reefs of Pohnpei, both Coleman et al. (2018) and Rocha et al. (2018) found shifts in species composition and abundance as depth increases, showing high abundances of serranid fishes at deeper depths. In general, *Liopropoma* are cryptic, and most species have their depth range spanning mesophotic depths (Randall and Taylor 1988, Baldwin and Robertson 2014). Therefore, continued exploratory work on mesophotic coral ecosystems is poised to reveal additional new species within this genus.

## Supplementary Material

XML Treatment for Liopropoma incandescens
